# Experiences of Clients and Professionals with the Recovery Oriented Intake

**DOI:** 10.1007/s10597-024-01250-1

**Published:** 2024-02-24

**Authors:** Fabiana Engelsbel, Nanette Waterhout, Marty Dijkstra, René Keet, Annet Nugter

**Affiliations:** 1 GGZ Noord-Holland-Noord Research Department, Heerhugowaard, The Netherlands; 2 GGZ Noord-Holland-Noord Department of Community mental Health, Heerhugowaard, The Netherlands; 3 GGZ Noord-Holland-Noord FIT-Academy, Heerhugowaard, The Netherlands

**Keywords:** Recovery, Recovery Oriented Intake, Peer Experts, Mental Health

## Abstract

The Recovery Oriented Intake (ROI) integrates recovery principles from the start of treatment, and involves peer experts, unlike the intake as usual (IAU). This study compared experiences with ROI and IAU among 127 clients and 391 professionals, consisting of practitioners and peer experts. Intake’s quality, measured with questionnaires, showed no differences in experiences between ROI and IAU clients. However, practitioners experienced ROI as more recovery-oriented than IAU. The ROI Fidelity Check (RFC) revealed that clients’ RFC-scores, but not practitioners’, predicted their valuation of intake’s quality. This underscores the need for (re)training and peer supervision for professionals to ensure adherence to ROI’s principles. Discrepancies between clients’ and professionals’ experiences at the start of treatment are consistent with literature on working alliance and Shared Decision Making (SDM). Differences between ROI and IAU professionals may stem from heightened awareness of recovery principles due to training and the presence of peer experts during intake.

## Introduction

When people with psychological problems seek help from a mental health institution, they often have specific expectations: they want to share their story, discuss with professionals about what could help them, collaborate in setting recovery goals, and know what they can expect from their treatment. These findings are based on a customer journey analysis conducted in 2016 (N. Kuijper & T. Dhondt, Personal communication, 2016), which mapped the application and intake process in mental health care. This analysis was the first step in the development of the Recovery Oriented Intake (ROI), designed to better match these recovery-oriented needs. This study is an important step in the (further) development of the ROI, as it aims to compare whether clients and professionals experience the ROI as more recovery oriented than the intake as usual (IAU).

Recovery-oriented care focuses on the strengths of clients and on leveraging the existing resources around the client, however big or small those resources may be. Recovery is the client’s journey, and the task of mental health professionals is to support this journey. This understanding of recovery comes from people with lived experiences, so-called peer experts, and is based on the idea that persons should be able to feel in control of their own lives, rather than simply doing what a health professional tells them to do (Rennick-Egglestone et al., 2020).

The recovery movement has had a great impact on mental health care (Slade et al., 2014 ). The focus in mental health care has shifted from merely remission of symptoms to a broader process of recovery, aspiring towards a life that is satisfying, fulfilling and enjoyable. In line with this movement, community mental health teams are now placing a greater emphasis on recovery of health, social functioning and personal identity (Slade, 2013). However, this recovery-focused approach has not been uniformly applied across all patient groups and treatment stages within mental health care. It is predominantly integrated into the care for persons with severe mental illness (SMI) of longer duration. Therefore, GGZ Noord-Holland-Noord (GGZ NHN), an integrated mental health service in the Netherlands, aimed to apply the principles of recovery in all aspects of treatment, including the initial phase – the intake. With the findings of our customer journey in mind, we designed the ROI, which is based on the five following principles: personal recovery, positive health, personal diagnostics, motivational interviewing and supported decision making (SDM).

The first principle, personal recovery, entails the restoration of a personal process. It involves rediscovering one’s own identity with a positive sense of self and constructing a meaningful life. Personal recovery is not solely about the complaints themselves, but rather about their consequences on a personal, social, psychological and physical level. Professionals support the process of recovery by providing clients with the help and treatment needed to pursue the life they want to lead, despite psychological problems (Van Weeghel et al., 2019; Shepherd et al., 2008; Slade et al., 2012). Recovery-oriented professionals emphasize strengths, goals and talents.

The second principle, positive health, refers to a broader perspective on health, moving away from the traditional notion of health as the absence of disease. This broader approach emphasizes individuals’ capacity to adapt and take control over their own lives whenever feasible, even in the presence of physical, emotional and social life challenges (Huber et al., 2011). The ability to adapt and take control can be enhanced by leveraging resources at the interpersonal level, such as social support and participation, and at the intrapersonal level, including perceived control, optimism, and personal mastery (Jeste et al., 2015). This concept is the core of a ROI: the conversation adopts a broader perspective on an individual’s resources across crucial life domains (e.g. daily functioning, social participation, mental well-being) and aims to collaboratively explore opportunities to strengthen the client’s autonomy.

The third principle emphasizes the client’s story and the reasons why the client sought help. Van Os refers to this as ‘personal diagnostics’, which revolves around four central questions: “What happened?”, “What are your vulnerabilities and your resilience?”, “Where do you want to go?”, and “What do you need?” (Longden, 2013; Van Os, 2018).

Motivational interviewing is the fourth principle and involves a collaborative, person-centered conversational style aimed at encouraging a client’s motivation to initiate and reinforce change (Miller & Rollnick, 2013). It is about understanding the client’s frame of reference and clarifying the client’s current behavior and goals, while emphasizing self-efficacy. This approach strengthens motivation for the chosen path to recovery.

Finally, supported decision making is a key principle encouraged in the ROI, which revolves around providing options and making decisions together. This principle aligns with the concept of shared decision making (Drake et al., 2010; Elwyn et al., 2012). It involves a collaborative approach in which the professional and the client jointly decide on the most appropriate follow-up, considering the available information on effective treatments and discussing alternatives to help clients evaluate different options. We prefer the term ‘supported decision making’ to emphasize our respect for the client’s decisions, even if they differ from the professional’s perspective (Pathare & Shields, 2012; Simmons & Gooding, 2017).

These five principles emphasize that the intake is not so much about the specific complaints, but it’s primary purpose is rather to explore an individual’s story and the broader context in which their symptoms and complaints arise. Consequently, the starting point is not the professional’s perspective of possible treatments, but the client’s needs, which may also imply that treatment might not be appropriate at all. This shift in perspective also transforms the role of the professional from an authority figure with expertise to an equal conversation partner. The professional places his er or her expertise next to the client’s, and offers support in making the most appropriate decision. This approach can only be realized when the contact between professional and client is characterized by equality. Diagnosis and choice of treatment evolve through a process of learning together (Koksma & Kremer, 2019), where the client’s own experiential knowledge and self-determination are always the starting points.

Peer experts play an important role in the ROI as allies of the client. They encourage hope, offer a perspective on life ‘beyond’ the disorder (Keet et al., 2019; Deegan, 2007; Farkas & Boevink, 2018), and support clients in their first steps towards this new phase. Peer experts bring valuable experiential knowledge, having navigated the challenges of mental dysregulation and the journey to recovery themselves. The presence and support of peer experts often lead to recognition and acknowledgment from clients. They also promote the process of supported decision making, as shown by a study involving young people in an intervention that combined peer work with shared decision making. Clients reported feeling more involved in their assessment and experiencing lower decisional conflict after engaging with a peer worker, which also led to increased client satisfaction (Simmons et al., 2017).

A meta-analysis showed that interventions where mental health professionals and peer experts collaborate can lead to increased personal recovery, empowerment and hope (Thomas et al., 2018). However, eliciting the personal perspective of clients, a crucial topic in person-centered care and SDM, is still rarely practiced in clinical settings (Rake et al., 2022). The inclusion of peer experts ensures the presence of three sources of knowledge: science, practice and lived experience. This comprehensive approach helps focus on all dimensions of recovery: symptomatic, social and personal.

The ROI was developed in 2017 at GGZ NHN in The Netherlands. Its first draft was designed by both practitioners and peer experts. We implemented the ROI in a step-by-step manner, starting in one of the three regions of the organization. This approach allowed us to compare the experiences of clients who attended a ROI with those of clients who underwent an IAU in the other two regions. A preliminary study focused on this comparison revealed no clear statistically significant difference between the two groups of clients in terms of their appraisal of the intake process (A. Nugter & F. Engelsbel, personal communication, 2022). The interpretation of this finding was seriously hampered by the lack of data about how the two types of intakes were actually conducted, such as with a fidelity measure. A fidelity measure did not exist: for the ROI we only had a preliminary description, and for the IAU no formal description or protocol existed at all, which was a remarkable discovery in itself. In addition, many clients who participated in a ROI also had a separate IAU, complicating the attributions of their evaluations specifically to the ROI. Thus we decided to repeat an improved version of this study.

A manual was written for the ROI including a ROI fidelity scale (Waterhout et al., 2022). This manual became the basis for the corresponding ROI training and was also used in supervision and peer supervision. In this way, the principles of the ROI were made explicit. Important parts of the manual and accompanying fidelity scale are: (1) The format of the ROI, which involves aspects such as a warm reception by the peer expert, location of the intake (if possible at the Recovery College), participants (including the client, a peer expert, one or two practitioners, a family member); (2) The content: what is discussed in the ROI, such as the personal story, the phase of recovery, the strengths and complaints, the needs of the clients and the possibilities within and outside mental health care; (3) The attitude of the clinicians and peer experts, which concerns aspects such as equality in contact and acknowledgment of the client’s direction and expertise.

This study aims to compare the client’s valuations of the quality of their intake (either the ROI or IAU), and to relate these valuations to the degree to which the intakes were performed in agreement with the ROI manual and fidelity scale.

The research encompasses two set of goals. Primarily, the aim of this study is to conduct a comprehensive evaluation of the extent to which the ROI adheres to its principles in practice. Secondly, the study aims to compare the perceived quality of intake experiences between clients who underwent the ROI and those who underwent an IAU. Based on insights gained from this research, we would like to propose practical suggestions for enhancing the implementation of the ROI.

To achieve these study aims, four questions have been formulated. The primary question in this study is: 1) Is there a difference in the perceived quality of the intake between clients who have experienced a ROI and clients who have experienced an IAU?

The following questions are secondary and focus on the extent to which the ROI adheres to its principles in practice from the viewpoint of both clients and professionals:

2a) Do participants (clients and professionals) in both the ROI and the IAU differ in their opinion on whether their intake adheres to the principles of the ROI (format, content and attitude)?

2b) Do clients and professionals differ in their opinion on whether the ROI was executed in line with its principles regarding content and attitude?

The final secondary question focuses on the predictability of clients’ valuation of their intake based on the degree to which their intake was conducted in accordance with the ROI principles:

2c) To what extent is the clients’ valuation of the intake predicted by the opinions of both clients and professionals about the performance of the intake?

Although we initially hypothesized that the ROI would be more favorably perceived than the IAU, we acknowledge an important caveat: we cannot rule out that over time some of the ROI-principles, such as ‘attention to personal recovery and positive health’ and ‘supported decision making’ might have been incorporated into the IAU as these have become increasingly commonplace in mental health care. The main distinction lies in the ROI, as these principles are not only operationalized, but are also integral to a training course, potentially making professionals more aware of them. Question 2b is exploratory: we do not have preconceived notions about the extent to which clients and professionals will similarly experience the ROI. With regard to question 2c, we expect that the experiences of clients are more predictive of their valuation than those of the professionals.

## Method

### Design

A cross-sectional comparative design in a naturalistic setting was used to assess the differences in intake experiences between participants of either the ROI or the IAU. Further, this study aimed to determine whether clients’ intake valuation can be predicted by their experiences regarding how the intake was conducted.

### Participants

Participants were clients, practitioners and peer experts. Clients between the ages of 18–65 who were referred for treatment to GGZ NHN, and offered an intake, were included in the study. In two of the three regions, clients underwent the ROI. In the other region, clients underwent the IAU. Clients were asked if they were willing to participate in an evaluative study of the intake. Participants were excluded if they were not fluent in Dutch and/or if they were not referred through the regular screening procedure.

In general, the mental health professionals who attended the ROI were a peer expert (PE), a coordinating practitioner (CP, mostly a master in psychology or a nurse) and a leading practitioner (LP, mostly a psychiatrist or clinical psychologist); in the IAU only the CP and LP participated.

To compute the required sample size in order to answer the hypotheses, the program GPower was used. The a priori calculated sample size for the first two hypothesis was *N* = 199 per condition (ROI vs. IAU), given that α = 0.05, statistical test = independent t-test, Cohen’s *d* = 0.25 and power = 0.80. For the third hypothesis the a priori calculated sample size was *N* = 207 per group (LP, CP, PE), given that α = 0.05, statistical test = ANOVA, Cohen’s *f* = 0.125 and power = 0.80. Since a participation rate of 60% was expected, a total of approximately 331 clients and 345 mental health professionals was required to attain adequate power to detect an effect of that size. For the fourth hypothesis we expected a larger effect size of Cohens *f*^2^ = 0.2, resulting in an a priori required sample size of *N* = 91, given that α = 0.05, statistical test = linear multiple regression, number of predictors = 10, and power = 0.80.

### Instruments

#### Quality of the Intake: The Clients’ Perspective

Clients’ valuation of the quality of the intake was measured on three dimensions. (I) ‘General Attitude of the Professional’, a subscale derived from the Consumer Quality Index (CQ-I; Linszen & de Beurs, 2018), consisting of 3 items answered on an adapted scale ranging from 1 ‘not at all’ to 10 ‘to a very high degree’, resulting in a 3–30 range of scores. (II) ‘Valuation of the Proposed Continuation of Care’ (“What grade would you give the choice you made?”) and (III) the ‘Overall Impression of the Quality of the Intake’ (i.e. “What is your overall impression about the quality of the intake?”) were both assessed with a one item Likert scale ranging from 1 ‘very bad’ to 10 ‘very good’.

#### The Performance of the Intake from Clients’ and Professionals’ Perspective: The ROI-Fidelity Check

On the basis of the ROI’s manual^1^, an instrument was developed with questions about the Format of the intake (9 items), Content of the intake (15 items), and the Attitude and Behavior during intake (7 items), the so-called ROI-fidelity check (RFC). The questionnaire’s content was elaborately discussed during our expert consultations, which included a peer expert, psychiatrist, researcher, and psychologist. To assess usability, the questionnaire was initially tested by clinicians and peer experts.

The items regarding the Format were questions about several factual aspects of the intake, such as “Was there a warm welcome prior to the intake?” and “Did you complete one or more questionnaires before the intake regarding your complaints/quality of life, either online or at-site?” which were answered with yes/no. Other factual multiple-choice questions allowed respondents to choose one or multiple options from a list of possible answers. For example, “How many individuals were present during the intake, excluding yourself?” (e.g. 1,2,3, etc.) and “Who were these individuals?” (e.g. psychiatrist, psychologist, etc.). The 15 items about the Content consist of statements related to what had been discussed during intake. Two of these 15 items were answered with yes/no, namely “A genogram is made of your family” and “The miracle question (i.e. how he/she envisions what his/her life would be like in a few years) has been addressed.” The remaining 13 items were answered on a 10-point Likert scale ranging from 1 ‘strongly disagree’ to 10 ‘strongly agree’. For example, “It has become clear what is meaningful to you”. The seven items related to Attitude consisted of statements about the manner in which the intake conversation was conducted, such as “There was equality in the interaction between you and the intake professionals”. Respondents provided answers on a 10-point Likert scale, ranging from 1 ‘strongly disagree’ to 10 ‘strongly agree’. Since a manual about how the IAU has to be performed, was absent, we decided to compare both types of intake only with the RFC.

In order to reduce dimensionality and to be able to compare the perspectives of the different types of participants, two separate factor analyses were conducted on the data of clients’ (N = 127) and practitioners’ (N = 307) RFCs. The data of all clients’ and all practitioners’ RFCs were used, including data of clients of whom we did not have the corresponding practitioners’ data and vice versa. A factor analysis was not conducted on the data of peer experts, because there was too little variation on the item level. The dichotomously scored items, as well as one item regarding the ‘Content’ (‘the four questions for personal diagnosis’) were not included in the (final) factor analyses. Reason for not including the Content item was that it did not load well on any of the factors. The factor analyses were conducted on data of the 18 clients’ and on the 17 practitioners’ version items, since the item “Client’s preferences were sufficiently discussed” was, by mistake, not included in the practitioners’ version.

Both analyses resulted in a comparable structure of the following three scales: 1) The scale ‘Client Central’ consisted of 10/11 items in the clients’/clinicians’ version respectively. Besides informing clients, most items focus on client’s ownership, wishes and client’s expertise. This scale had a good internal consistency of 0.85 (Cronbach’s alpha) for both groups. The two remaining scales contained less items: 2) the scale ‘Equality’ (3 items) measures the equality in the contact between client and professionals and between professionals and also had a good internal consistency with a Cronbach’s alpha of 0.85 for both groups. 3) The scale ‘Positive Health’ consisted of three items on promoting and impeding factors to mental health, and all aspects of client’s mental health. For the clients’ version the Cronbach’s alpha was 0.70 (acceptable) and for practitioners 0.795 (almost good).

### Intervention

Clients were invited for an intake after a first online screening and after filling out Routine Outcome Monitoring (ROM) questionnaires. This part of the procedure was the same for both the ROI and IAU participants. The ROI took place preferably at the Recovery College (i.e. a place where peer experts support clients in their recovery with several (self-help) programs and groups), but could also take place at the location of clients’ team. It was standard to invite a close relative or friend of the client, always in agreement with the client. Before the start of the intake, the client was welcomed by a peer expert in order to make the client feel comfortable. The total intake duration was approximately 45 min. The primary goal was to conduct a thorough assessment of client’s request for help, and to inventory what the client needed to gain better control over his/her own recovery process. After the ROI, there was an opportunity for a brief follow-up discussion with the peer expert, e.g. to discuss how the client experienced the intake or to address any remaining questions.

### Procedure

Upon registration, clients who granted permission to be approached for participating in a research study were compiled into a list by screeners. This list was securely stored in a central location within a protected folder that could be accessed by the researcher. Only clients included in this list were contacted for participation in the current study.

On a weekly basis a printout was made of the intakes that had taken place in the penultimate week. Thus, in week five a printout of week three was made. This two-week latency was due to the finalization process of the contacts and ‘no shows’, which lasts two weeks. The maximum duration between the intake and the submission of the questionnaire was set on six weeks.

Subsequently, the RFC was being prepared for the intake professionals, who received an email with the request to fill in the RFC for each of the clients separately. If the RFC was not submitted within a week, a friendly reminder was send by mail.

Clients were contacted by phone during which the RFC and the questionnaire about the Quality of the Intake was conducted by the researcher. Also, clients’ permission for enabling the usage of data for research and scientific publication was asked.

Approval for the procedure was granted by the internal scientific committee of the organization and the privacy protocol of the organization was followed.

### Data-Analyses

To test if there was a difference in opinion about the intake between clients who experienced a ROI or IAU, multiple independent t-tests were performed for items that were normally distributed, or Mann-Whitney tests for items that were not normally distributed. A Bonferroni correction was applied, resulting in an α = 0.01.

To test if ROI and IAU participants (clients and professionals separately) differed in their opinion whether their intake was performed according to the principles of the ROI, regarding Format, Content, and Attitude, multiple t-tests were performed for normally distributed items, or Mann-Whitney tests for not normally distributed items. A Bonferroni correction was applied for the 8 Format items, resulting in an α = 0.0063. For the RFC subscales (3 in total) the Bonferroni correction resulted in an α = 0.0167.

To test if clients and professionals differed in their opinion on whether their intake was performed according the principles of the ROI, regarding Content and Attitude, several Kruskal-Wallis tests were conducted. A Bonferroni correction was applied, resulting in an α = 0.0167.

For all analyses involving the professionals, separate analysis were conducted for LPs, CPs, and PEs. This approach was necessitated by variations in the availability of scores for each intake. In some instances evaluations from all three perspectives (LPs, CPs, and PEs) were available, while for other intakes, evaluations were limited to only one perspective, either LPs, CPs, or PEs. Consequently, only one perspective per intake was incorporated into the analyses to ensure a valid comparison. However, no adjustments were made to deal with the clustering of the professionals’ data due to the difficulty in accurately determining the total number of distinct professionals who completed the RFC. Despite professionals providing their names in the questionnaire, the variability in name entries made it challenging to reliably group responses by individual professionals.

Finally, in order to examine if the ‘Overall Impression of the Quality of the Intake’ was predicted by clients’ and professionals’ RFC, four separate multiple regression analyses were performed with the ‘Valuation of the Intake’s Quality’ as the dependent variable, using the ENTER method. In the first analysis 7 (out of 9) Format items were included as predictors as well as the 3 clients’ RFC subscales, and in the following three analyses the same predictors as beforementioned including the RFC subscales of one of the intake professionals (LP,CP, PE).

## Results

### Number of Participants

The study took place from January 2021 till the end of August 2021. During this period we were able to approach *N* = 250 clients who were willing to participate in the study of which *N* = 110 had been subjected to the ROI and *N* = 140 to the IAU. Of these 250 clients eventually *n* = 127 clients (50.1%) participated in the study of which *n* = 57 with a ROI and *n* = 70 with an IAU. In merely all cases the reason of drop-out was that the telephonic interview couldn’t take place within six weeks after the intake. The mean duration between the ROI date and the interview date was 4.11 weeks (SD = 1.62), and for the IAU 4.6 (SD = 1.63). For 21 clients the duration exceeded the six weeks, and four clients weren’t able to answer the questions due to an insufficient fluency in Dutch.

The total number of intakes for which professionals completed the questionnaire, either for the ROI or the IAU, was *n* = 391. The distribution per type of professional is provided in Table 1. It is worth noting that the numbers in Table 1 represent the count of intakes, not the count of individual professionals. Within each type of professional, there may be instances where some individuals are the same person. Of the 57 participating ROI clients, *n* = 127 professionals had completed the questionnaire, and of the 70 participating IAU clients this number was *n* = 71. Of the 123 clients who did not participate in the study *n* = 119 ROI professionals and *n* = 74 IAU professionals completed the questionnaire. The mean duration between the intake data and completion of the questionnaire was 2 weeks (SD = 1.04) for the peer experts, 2.44 (SD = 0.72) weeks for the CPs and 2.68 (SD = 1.31) weeks for the LPs.

**Table 1:** The number of intakes for which professionals completed the questionnaire for the recovery oriented intake (ROI) and intake as usual (IAU) separately

Type of professionals	ROI (*n* = 57 clients)	IAU (*n* = 70 clients)	ROI clients who did not participate (*n* = 53 clients)	IAU clients who did not participate (*n* = 70 clients)
Leading Practitioner	*n* = 35^*^	*n* = 38^*^	*n* = 31^*^	*n* = 40^*^
Coordinating practitioner	*n* = 46^*^	*n* = 33^*^	*n* = 46^*^	*n* = 34^*^
Peer expert	*N* = 46^*^		*n* = 42^*^	
Number of professionals per intake type	*n* = 127^*^	*n* = 71^*^	*n* = 119^*^	*N* = 74^*^

* The numbers in the Table represent the count of intakes, not the count of individual professionals

### Experiences with the Intake: A Comparison Between the ROI and IAU Clients

In this subsection the results are presented regarding the difference in valuation about the quality of the intake between clients who experienced a ROI versus an IAU. The mean scores and standard deviations of the ROI and IAU clients on the two dimensions regarding the experiences with the intake can be found in Table 2. There were no statistically significant differences in scores between the ROI (MeanRank = 64.91) and IAU clients (MeanRank = 63.26) regarding the ‘General Attitude of the Professional’ (*U* = 2047.000, *z* = 0.254, *p* = .800) and the ‘Overall Impression of the Intake’s Quality’ (ROI MeanRank = 65.88; IAU MeanRank = 62.47; *U* = 2102.000, *z* = 0.534, *p* = .593). The presence of the peer expert was valued by ROI clients with a mean of 8.04 (SD = 2.03).

**Table 2:** Mean scores (M) and standard deviations (SD) on the three dimensions regarding experiences with the intake

Valuation	ROI M (SD)*	IAU M (SD)*
*Whole group*	*n* = 57	*n* = 70
General attitude of professional	25.28 (4.682)	25.34 (4.303)
Overall impression	7.82 (1.692)	7.70 (1.697)

* Because ranks do not represent the actual values, the M and SD are included in the Table. The analyses were done on the mean ranks, which are reported in the text

### ROI Fidelity Check: A Comparison in Performance Between the ROI and IAU for Clients and Professionals Separately

#### Clients

In this subsection, the results describe whether participants in the ROI and the IAU (clients and professionals) differed in their opinions regarding the extent to which the ROI principles were upheld during the intake. On 2 of the 3 Format items statistically significant differences were found between the ROI and IAU clients: relatively more ROI (87.7%) than IAU (57.1%) client answered ‘yes’ on the question if they had a warm welcome (*p* < .001), and almost all ROI clients (87.7%) versus almost none of IAU clients (2.9%) answered ‘yes’ on the question if they were welcomed by and received some information of a peer expert (*p* < .001). Regarding the question if the scores of the ROM and screening questionnaires were discussed with them, relatively more IAU (58%) than ROI clients (42%) answered ‘yes’, but after the Bonferroni correction this difference was not statistically significant (*p* = .022).

The mean, standard deviation, *p*-values, and/or mean rank for each of the RFC subscales are presented in Table 3, for each of the ROI and IAU participants. On none of the three RFC subscales – ‘Client Central’, ‘Equality’ and ‘Positive Health’ – the scores showed statistically significant differences between the ROI and IAU clients (‘Client Central’: *t*(125) = − 0.538; ‘Equality’: *U* = 2012.500, *z* = 0.085; ‘Positive Health’ *U* = 1761.000, *z* = -1.138).

**Table 3:** Mean (M), standard deviation (SD), mean rank (MR) and p-values for each RFC subscale, for the ROI and IAU participants separately

	ROI M** (SD)	ROI MR	IAU M** (SD)	IAU MR	P-value
*Clients*	*n* = 57		*n* = 70		
Client Central	57.56 (19.83)		55.80 (16.55)*		0.591
Equality	23.68 (5.62)	64.31	23.69 (5.69)	63.75	0.932
Positive Health	19.70 (6.32)	59.89	21.01 (5.71)	67.34	0.255
*Leading practitioners*	*n* = 35		*n* = 38		
Client Central	73.54 (8.29)	46.04	63.76 (12.41)	28.67	
Equality	23.49 (2.79)	33.36	24.53 (2.98)	40.36	0.139
Positive Health	23.00 (2.36)	40.29	22.47 (2.86)	39.59	0.480
*Coordinating practitioners*	*n* = 46		*n* = 33		
Client Central	76.78 (9.93)		68.18 (11.92)		
Equality	24.69 (3.18)	42.09	23.79 (4.60)	37.09	0.336
Positive Health	23.07 (3.96)	40.29	23.24 (2.76)	39.59	0.892
*Peer experts*	*n* = 46				
Client Central	78.54 (8.96)				
Equality	25.11 (2.74)				
Positive Health	24.02 (2.97)				

* The mean of this subscale is calculated with one item less in order to make the clients’ mean comparable with that of the professionals

** Because ranks do not represent the actual values, the M and SD are also included in the Table. Most analyses were done on the mean ranks

On the Content item that was not included in the three subscales of the RFC, the scores showed statistically a significant difference: ‘the four questions for personal diagnosis’ were more central during the ROI (MeanRank = 75.86) than during the IAU (MeanRank = 54.34; *U* = 2671.000, *z* = 3.332, *p* < .001).

#### Practitioners

The LPs who conducted a ROI had statistically significant higher scores than the LPs who conducted an IAU on the subscale ‘Client Central’ (*U* = 981.500, *z* = 3.499) but not on the subscales ‘Equality’ (*U* = 537.500, *z* = -1.480) and ‘Positive Health’ (*U* = 728.000, *z* = 0.707). These same results apply also to the scores of the CPs (‘Client Central’: *t*(77) = -3.585) (‘Equality’: *U* = 855.000, *z* = 0.962) (‘Positive Health’: *U* = 772.500, *z* = 0.135, see Table 3).

### ROI Fidelity Check: A Comparison in ROI Performance between Professionals and Clients

#### Clients versus Professionals

In this subsection, the results present a comparison between clients’ and professionals’ perspectives on whether the ROI is performed according to its principles. To enable a comparison between clients and professionals, a custom total score for clients was calculated, excluding the item omitted in the professionals’ version. There are data of 35 clients and LPs, 46 clients and CPs and 46 clients and PEs. The mean scores/ranks on each of the RFC subscales are shown for each group of intake professionals in Table 4.

**Table 4:** Mean (M), standard deviation (SD), mean rank (MR) and p-values of clients and professionals for each RFC subscale

Subscales RFC	Professional Mean (SD)	Professional MR	Clients Mean (SD)	Clients MR	P-value
*Leading Practitioners vs. Clients (n = 35)*					
Client Central	73.54 (8.29)		56.94 (17.24)		
Equality	23.49 (2.79)	35.20	22.83 (5.81)	35.80	0.901
Positive Health	23.00 (5.59)	42.66	19.69 (5.51)	28.34	0.003
*Coordinating Practitioners vs. Clients (n = 46)*					
Client Central	76.78 (9.39)		56.57 (20.91)		
Equality	24.96 (3.18)	48.28	23.52 (5.83)	44.72	0.520
Positive Health	23.07 (3.96)	54.77	19.46 (6.40)	38.23	0.003
*Peer experts vs. Clients (n = 46)*					
Client Central	78.54 (8.96)		59.39 (19.76)		
Equality	25.11 (2.74)	47.04	24.39 (4.78)	45.96	0.844
Positive Health	24.02 (2.97)	56.85	19.87 (6.27)	36.15	

The scores on the subscale ‘Equality’ are not statistically significant different between clients and LPs (*U* = 602.000, *z* = − 0.124), but for the subscales ‘Client Central’ (*t*(48.934) = -5.133) and ‘Positive Health’ (*U* = 863.000, *z* = 2.968) they are (see Table 4). On both scales clients scored lower than LPs. The same results apply for the comparison between clients and CPs (‘Client Central’: *t*(62.439) = -5.982; ‘Equality’: *U* = 1140.000, *z* = 0.643; ‘Positive Health’: *U* = 1438.500, *z* = 2.982) and for the comparisons between clients and peer experts (‘Client Central’: *t*(156) = -5.986; ‘Equality’: *U* = 1083.000, *z* = 0.197; ‘Positive Health’: *U* = 1534.000, *z* = 3.740).

### Predictors of the Valuation of the Intake’s Quality

Apart from the three subscales of the RFC, the following Format items were included as predictors in all four multiple regression analyses: (1) “Did you follow the module ‘mapping your problem, recovery in sight’ prior to your intake?” (2) “Did you complete at least one questionnaire, online or on location, about your complaints/quality of life prior to your intake?” Both items were rescored to a dichotomous yes/no (1/0) scale. (3) “Was there a warm welcome?” (4) “Was there a short follow-up discussion with you?” (5) “The wonder question was asked.” (6) “A genogram was made of your family.” And finally (7) “Were you welcomed by and received some information from a peer expert?” All these items were dichotomously answered with yes (1) or no (0).

Only the overall regression (*N* = 127), which included clients’ RFC subscales and Format items significantly predicted the valuation of the intake’s quality (R^2^ = 0.507; *F*(3,123) = 42.112, *p* < .001). It was found that ‘Client Central’ (β = 0.293) and ‘Equality’ (β = 0.473) were statistically significant predictors of this valuation (*t*(126) = 3.693, *p* < .001 and *t*(126) = 6.208, *p* < .001 resp.). As the score on ‘Client Central’ increased by 1 point, the valuation of the Intake’s Quality also increased by 0.3 points. Similarly, for every one-point increase on ‘Equality’, the valuation increased by over 0.4 points.

## Discussion

This study represents a significant step in the development of an intake process that focuses on recovery and positive health. An important step in this process was the operationalization of ROI principles through the RFC, which includes two versions: one for the client and one for the professionals. Analyses revealed a highly comparable and reliable factor structure for both clients and practitioners, consisting of three factors that define the ROI’s main focus: ‘Client Central’, emphasizing the client’s ownership, wishes and needs, and expertise; Equality in the contact between clients and professionals with professionals acting as allies rather than authorities; and with an emphasis on ‘Positive Health’. With this scale we have the tools to further study and improve the ROI.

The central questions of the study concerned (1) whether clients perceived the ROI as an improvement compared to the IAU, and (2) whether both clients and professionals experienced the ROI more as a method in which the needs and wishes of the client are central to the conversation, and in which the expertise of clients and professionals is considered equal.

The findings revealed no differences between the experiences of clients who underwent a ROI and those who had an IAU. The general valuation of both types of intakes by clients was relatively positive, with mean scores of 7.8 and 7.7 on a scale of 1 to 10, respectively. Contrary to our expectations, clients did not perceive the ROI as more client-centered, more focused on positive health, or involving more equality in contact with the professional compared to the IAU. However, differences were noted in two aspects of the format and one aspect of the content concerning personal diagnostics, which we had hypothesized. These format aspects include the warm welcome and the information provided by the peer expert before the intake, as explicitly stated in the ROI manual. Since peer experts do not participate in the IAU, it is not surprising that a larger number of ROI clients experienced this welcome compared to IAU clients. Similarly, more clients in the ROI group reported the application of personal diagnostics, as detailed by Van Os (2018) and emphasized in the ROI manual, compared to those in the IAU group. Additionally, all professionals perceived the ROI as being more focused on positive health and more client-centered in comparison to the clients’ perspectives.

The lack of significant differences in the experiences of the two client groups may suggest that both types of intake are more similar than we expected. This similarity could be partly due to the existing orientation towards positive health and recovery in the organization and within mental health care in the Netherlands in general, where the involvement of peer experts is not a novel concept, and knowledge and experiences are widely shared among professionals. Alternatively, the lack of differences may stem from the fact that for many clients, the intake is the first contact with mental health care. This might make them less sensitive for the probably subtle differences in approaches compared to clients already undergoing treatment. It is also important to note that the study did not include as many clients as needed according to the power analyses, which is a significant limitation. However, the observed differences between clients were so marginal that a much larger sample would be required to achieve statistically significant differences. This raises questions about the clinical significance of any potential differences. Another limitation is the naturalistic design; clients were not randomly assigned to either the IAU or the ROI. Without random assignment, there is a potential for confounding variables that could have affected the observed outcomes, limiting the ability to draw firm conclusions about the causal relationship between the ROI and the observed outcomes.

The fact that professionals did experience differences might be attributed to the training received by those in the ROI. This training could have increased their awareness of the ROI principles. Additionally, the presence of the peer expert during the intake and the nature of questions they pose may continually remind practitioners of the ROI’s principles.

Clients not only perceived the intake as less client-centered and less focused on positive health than professionals did, but, as depicted in Table 4, clients’ responses exhibited much more variation compared to those of professionals. This suggests that professionals operate from a common frame of reference, whereas clients do not. These findings may indicate that during this initial contact, clients and professionals respond from distinct perspectives, posing a challenge for professionals to accurately assess and adjust their approaches to align with clients’ needs. This dynamic might change during treatment. The divergence in experiences between clients and professionals is also found in studies on the working alliance (e.g. Benthem et al., 2020) and SDM (e.g. Drivenes et al., 2020). Further, studies suggest that this alignment improves during treatment (Jennissen et al., 2020). Thus, it seems likely that the alignment between clients’ and professionals’ experiences in areas such as shared decision-making, working alliance, and the recovery-oriented approach is an ongoing and dynamic process. It involves continuous communication, collaboration, and adaptation over the course of the therapeutic relationship, rather than being limited to a single consultation. Alternatively, the reduced variation within the professionals’ data could stem from underestimation due to the interdependence within this dataset. As the same professionals administered the RFC for various clients, it’s reasonable to assume that scores assigned by the same professional would exhibit higher correlations with each other compared to those assigned by different professionals. This underestimation of the scores’ variability, and thus the standard error, could lead to an inflation of the test statistic and, consequently, resulting in a higher rate of Type I errors than specified. This may cause the observed differences between clients and professionals to be potentially overestimated.

The clients’ scores on the subscales ‘Client Central’ and ‘Equality’ of the client were predictive of their general valuation of the intake, emphasizing the importance of these principles of the ROI. Interestingly, the professionals’ scores on these factors were not predictive for the clients’ valuation of the intake, which is in line with the finding that scores of the clients and professionals differed. This result suggests that an increased adherence to ROI principles - as perceived by clients – is positively associated with their satisfaction with the intake. This finding aligns with the SDM literature (Carrotte et al., 2021; Loh et al., 2007). While no differences were observed between the ROI and the IAU, the guiding principles of the ROI appear to play a crucial role in patient satisfaction with the intake. This underscores the importance of further implementation of the ROI principles and guidelines in mental health care. Consequently, it is advisable to provide (re)training and peer supervision for professionals, along with regular checks to ensure that intakes are conducted in accordance with the guidelines outlined in the ROI manual.

In this study we were not able to explore to what extent the presence of the peer expert is essential for a ROI, although, as we stated before, it could quite well be that their presence is a constant reminder of the principles of the ROI, especially with regard to the focus on recovery and supported decision making. Also, their presence corresponded with the relative positive experience of the warm welcome of the clients who had a ROI. In general, the presence of the peer expert was experienced as very positive by the clients: they rated the presence of the peer experts with an 8. This is in line with the findings regarding peer support in mental health care which indicates that clients feel understood by peer experts and experience contact with them as equal (Repper & Carter, 2011).

In the organization where this study was conducted, both a LP and a CP were involved in conducting intakes, primarily to incorporate diverse perspectives on client’s problems. We do not know to what extent the presence of both types of practitioners is necessary in the ROI, as peer experts also offer a unique perspective.

The focus of this study was primarily on the clients’ general evaluation of the intake process. While understanding clients’ experiences with the intake is crucial for its further improvement, an interesting next step would be to examine how the emphasis on recovery during the ROI is reflected in the documentation of the intake process. Specifically, we plan to examine whether there is a difference between the ROI and IAU in the thoroughness of assessing all recovery domains and the integration of this written information into clients’ case conceptualization. Given that the quantity and quality of information collected during the intake influences diagnostic outcomes, case conceptualization, and the subsequent treatment, we will also explore if the type of intake has long-term effects. Our hypothesis is that participating in a ROI may facilitate quicker entry into recovery treatment, which may result in shorter treatment durations. Additionally, we are interested in comparing treatment outcomes in terms of recovery between the two groups. These questions will be addressed in follow-up studies.

## Conclusions

The aim of this study was to evaluate the ROI by comparing the experiences of the ROI and IAU participants, in terms of the quality of their intake and the extent to which their intake was performed in adherence to the principles of the ROI. Although experiences did not significantly differ between clients attending the ROI and those with an IAU, the general valuation of their intake increased as they also rated the adherence to the ROI principles higher. This finding underscores the importance of further implementation of the ROI guidelines in mental health care. This could involve offering (re)training and multidisciplinary peer supervision for professionals. The exchange of insights and experiences during peer supervision can foster a deeper understanding of the ROI principles and provide practical strategies for their effective integration into mental health care practices. Finally, to ensure the fidelity of ROI principles, it is advisable to conduct an annual recurring fidelity check.

Practitioners experienced the ROI as more client centered than the IAU. This perception is likely influenced by the training they received for the ROI and the presence of the peer expert during the intake, both of which probably enhance practitioners’ awareness of the ROI principles. For clients, however, the intake often represents their first contact with specialized mental health care, which may make them less familiar with concepts of recovery and positive health, and thus less perceptive for subtle differences in approach. Further, the ROI professionals experienced the intake as more client-centered and focused on positive health compared to the clients’ perspectives. This difference at the start of treatment aligns with research on working alliance and SDM. Recognizing this difference, and the option to address it, could enhance the communication between clients and professionals in subsequent stages.

## Footnotes

1 The manual outlines the procedural aspects of the ROI, detailing its structure (form), emphasizing the equal and compassionate approach (attitude) among the client and multiple professionals, and highlighting the focus on exploring the patient’s narrative and needs (content).

## References

[CR2] Carrotte, E. R., Hartup, M. E., Lee-Bates, B., & Blanchard, M. (2021). I Think That Everybody Should Be Involved: What Informs Experiences of Shared Decision-Making in Supporting People Living with Schizophrenia Spectrum Disorders? *Patient Education and Counseling*, *104(7)*, *1583–1590*. doi: 10.1016/j.pec.2020.11.012. Epub 2020 Nov 11. PMID: 33229188. 10.1016/j.pec.2020.11.012 33229188

[CR3] Deegan PE (2007). The lived experience of using psychiatric medication in the recovery process and a shared decision-making program to support it. Psychiatric Rehabilitation Journal.

[CR4] Drake RE, Deegan PE, Rapp C (2010). The promise of shared decision making in mental health. Psychiatric Rehabilitation Journal.

[CR5] Drivenes K, Haaland V, Hauge YL, Vederhus JK, Irgens AC, Solli KK, Regevik H, Falk RS, Tanum L (2020). Discrepancy in ratings of shared decision making between patients and health professionals: A cross sectional study in mental health care. Frontiers in Psychology.

[CR6] Elwyn G, Frosch D, Thomson R, Joseph-Williams N, Lloyd A, Kinnersley P, Cording E, Tomson D, Dodd C, Rollnick S, Edwards A, Barry M (2012). Shared decision making: A model for clinical practice. Journal of General Internal Medicine.

[CR7] Farkas M, Boevink W (2018). Peer delivered services in mental health care in 2018: Infancy or adolescence?. World Psychiatry.

[CR8] Huber M, Knottnerus JA, Green L, Van der Horst H, Jadad AR, Kromhout D, Leonard B, Lorig K, Loureiro MI, Van der Meer JWM, Schnabel P, Smith R, Van Weel C, Smid H (2011). How should we define health?. British Medical Journal.

[CR9] Jennissen S, Nikendei C, Ehrenthal JC, Schauenburg H, Dinger U (2020). Influence of patient and therapist agreement and disagreement about their alliance on symptom severity over the course of treatment: A response surface analysis. Journal of Counseling Psychology.

[CR10] Jeste DV, Palmer BW, Rettew DC, Boardman S (2015). Positive psychiatry: Its time has come. Journal of Clinical Psychiatry.

[CR11] Keet R, de Vetten-Mc Mahon M, Shields-Zeeman L, Ruud T, Van Weeghel J, Bähler M, Mulder CL, Van Zelst C, Murphy B, Westen K, Nas C, Petrea I, Pieters G (2019). Recovery for all in the community; position paper on principles and key elements of community-based mental health care. Bmc Psychiatry.

[CR12] Koksma JJ, Kremer JAM (2019). Beyond the Quality Illusion: The learning era. Academic Medicine.

[CR13] Linszen DFE, de Beurs E (2018). Patient experience as measured with the brief CQ-I: An evaluation. European Journal for Person Centered Healthcare.

[CR14] Loh, A., Simon, D., Wills, C. E., Kriston, L., Niebling, W., & Härter, M. (2007). The Effects of a Shared Decision-Making Intervention in Primary Care for Depression: A Cluster-Randomized Controlled Trial. *Patient Education and Counseling*, *67(3)*, *324 – 32*. 10.1016/j.pec.2007.03.023. Epub 2007 May 16. PMID: 17509808. 10.1016/j.pec.2007.03.023 17509808

[CR15] Longden, E. The voices in my head, Kindle edition, TED conferences, Long Beach, California, United States, 28-02-2013. https://www.youtube.com/watch?v=syjEN3peCJw.

[CR16] Miller, W. R., & Rollnick, S. (2013). *Motivational interviewing: Helping people change* (3rd ed.). Guilford Press.

[CR17] Pathare S, Shields LS (2012). Supported decision-making for persons with Mental illness: A review. Public Health Reviews.

[CR18] Rake EA, Box ICH, Dreesens D, Meinders MJ, Kremer JAM, Aarts JWM, Elwyn G (2022). Bringing personal perspective elicitation to the heart of shared decision-making: A scoping review. Patient Education and Counseling.

[CR19] Rennick-Egglestone S, Elliott R, Smuk M, Robinson C, Bailey S, Smith R, Keppens J, Hussain H, Pollock K, Cuijpers P, Llewellyn-Beardsley J, Ng F, Yeo C, Roe J, Hui A, Van Der Krieke L, Walcott R, Slade M (2020). Impact of receiving recorded mental health recovery narratives on quality of life in people experiencing psychosis, people experiencing other mental health problems and for informal carers: Narrative experiences Online (NEON) study protocol for three randomised controlled trials. Trials.

[CR20] Repper J, Carter T (2011). A review of the literature on peer support in mental health services. Journal of Mental Health.

[CR21] Shepherd, G., Boardman, J., & Slade, M. (2008). Putting recovery into mental health practice. *Mental Health Today*, 28–31. 18584845

[CR23] Simmons M, Gooding P (2017). Spot the difference: Shared decision-making and supported decision-making in mental health. Irish Journal of Psychological Medicine.

[CR22] Simmons MB, Batchelor S, Dimopoulos-Bick T, Howe D (2017). The Choice Project: Peer workers promoting Shared decision making at a Youth Mental Health Service. Psychiatric Services.

[CR24] Slade, M. (2013). *). 100 ways to support recovery: A guide for mental health professionals* (2nd ed.). Rethink Mental Illness.

[CR26] Slade M, Williams J, Bird V, Leamy M, Le Boutillier C (2012). Recovery grows up. Journal of Mental Health.

[CR25] Slade M, Amering M, Farkas M, Hamilton B, O’Hagan M, Panther G, Perkins R, Shepherd G, Tse S, Whitley R (2014). Uses and abuses of recovery: Implementing recovery-oriented practices in mental health systems. World Psychiatry.

[CR27] Thomas EC, Despeaux KE, Drapalsky AL, Bennet M (2018). Person-oriented recovery of individuals with serious mental illnesses: A review and meta-analysis of longitudinal findings. Psychiatric Services.

[CR1] van Benthem P, Spijkerman R, Blanken P, Kleinja M, Vermeiren RJM, Hendriks VM (2020). A dual perspective on first session therapeutic alliance: Strong predictor of youth mental health and addiction treatment outcome. European Child & Adolescent Psychiatry.

[CR31] Van Os, J., & De (2018). DSM-5 voorbij! Persoonlijke diagnostiek in een nieuwe ggz. *Bohn Stafleu Van Loghum: Houten the Netherlands*. 10.1007/978-90-368-2036-3. 1st ed.

[CR32] Van Weeghel J, Van Zelst C, Boertien D, Hasson-Ohayon I (2019). Conceptualizations, assessments, and implications of personal recovery in mental illness: A scoping review of systematic reviews and meta-analyses. Psychiatric Rehabilitation Journal.

[CR34] Waterhout N, Dijkstra M, Nugter A, Keet R, Van Rooijen S (2022). HOI! Niet Zo maar een begroeting. Ervaringen met De Herstel ondersteunende. Participatie en Herstel.

